# Effect of Nano-Filled Protective Coating and Different pH Enviroment on Wear Resistance of New Glass Hybrid Restorative Material

**DOI:** 10.3390/ma14040755

**Published:** 2021-02-05

**Authors:** Sandra Brkanović, Ana Ivanišević, Ivana Miletić, Dražen Mezdić, Silvana Jukić Krmek

**Affiliations:** 1 School of Dental Medicine, University of Zagreb, Gundulićeva 5, 10000 Zagreb, Croatia; sandra.brkanovic@gmail.com (S.B.); miletic@sfzg.hr (I.M.); jukic@sfzg.hr (S.J.K.); 2 Faculty of Mechanical Engineering and Naval Architecture, University of Zagreb, Lučićeva 5, 10000 Zagreb, Croatia; drazen.mezdic@fsb.hr

**Keywords:** glass ionomer, wear resistance, acid load, artificial saliva

## Abstract

The purpose of the study was to determine the wear rate of Equia Forte HT Fil with Equia Forte Coat or without coating and compare it with Fuji IX GP high-viscosity glass ionomer cement (GIC) in conditions with acid load or at neutral pH. The samples were stored for 7 days: (1) in artificial saliva, (2) in artificial saliva and cyclically exposed to low pH, and (3) in distilled water and cyclically exposed to low pH. Wear was determined by measuring the difference in mass before and after brushing in an abrasion testing device. The wear of Fuji IX GP was significantly higher than that of Equia Forte HT Fil with or without coating (*p* = 0.000). The difference between Equia Forte HT Fil with and without Coat was not statistically significant (*p* < 0.803). The differences in wear resistance between samples stored in saliva and in distilled water were not significant (*p* = 0.588). Periodic exposure to the low pH solution significantly affected the wear resistance of all materials (*p* = 0.000). Equia Forte HT Fil was more resistant to wear than Fuji IX GP in all storage conditions. A resinous coat did not significantly increase wear resistance.

## 1. Introduction

Glass ionomer cement (GIC) was invented in 1969 by Wilson and Kent, followed by McLean and Wilson introducing it to dentistry in the 1970s [1]. It is a two-component system based on a powder and a liquid formula. The powder contains calcium fluoroaluminosilicate glass particles, and the liquid is an aqueous solution of a homopolymer of polyacrylic acid or a copolymer of polyacrylic and maleic or itaconic acid [2,3,4]. Conventional GIC is set by an acid–base reaction; that is, when mixing powder and liquid, the organic acid in the liquid reacts with oxides from the powder (which act as proton acceptors), and acrylate salts and water are formed in the neutralization reaction [2]. GIC is a bioactive material that chemically bonds to hard dental tissues by ionic bonding mechanisms. In addition, the fluorine in the composition of these materials promotes remineralization and the cessation of carious lesions [2,5]. Due to a number of qualities—such as biocompatibility and low toxicity, good adhesion to enamel and dentin, pulp protection, reduced volumetric shrinkage, and the release of fluoride ions (i.e., caries-protection)—glass ionomer cements are used in wide applications in dental medicine [6,7]. The main disadvantage of GICs is their lower mechanical properties compared to composite resin restorative materials, which makes their use limited in areas of greater chewing stress [8]. Therefore, conventional GIC in posterior teeth is mostly used as a material for temporary restoration, especially in multiple-surface restorations [9,10].

Contemporary dentistry is based on the principles of minimally invasive approach, application of biomimetic materials, and the regeneration of dental tissues including tissue engineering [7,11,12]. Recent studies reported promising results in the regeneration of oral tissues using stem cells, including mineralized dental tissues. In parallel with advances in tissue engineering, progress has been made in the field of mechanical and biomimetic properties of restorative materials [13,14]. In the last 10–15 years, new materials derived from high-viscosity glass ionomers have gained their place in restorative dentistry. These materials have improved mechanical properties, and studies have shown that in certain clinical situations, they may/can be the material of choice for long lasting fillings in the posterior region [13,14]. In 2007, GC Corporation (Tokyo, Japan) has introduced a new restorative concept consisting of two components: Fuji IX GP Extra (GC, Tokyo, Japan) high-viscosity glass ionomer and nano-filled coating, which was later renamed Equia Fil. Better mechanical properties are achieved with a protective coating filled with nanoparticles. It is partially incorporated into the material, which in addition to mechanical enhancements, further improves the aesthetic properties of the material (using a microlaminated technique). Equia Forte Fil glass hybrid materials (GC, Tokyo, Japan) were developed on the Equia Fil platform in 2015, and the latest Equia Forte HT Fil material (GC, Tokyo, Japan) was produced in 2019. Glass hybrid materials based on GIC technology have been modified with glass particles of different sizes, such as highly reactive small particles being added to the standard filler. This property increases the reactivity and, according to the manufacturer, significantly affects the mechanical properties of the material and makes them suitable for long-lasting fillings in the posterior teeth [15,16]. The glass hybrid GICs also contain a nano-filled light-curing coating Equia Forte Coat (GC, Tokyo, Japan).

The glass hybrid material Equia Forte HT Fil and the high-viscosity GIC Fuji IX GP are clinically applied for long-lasting permanent restorations [17,18]. Hardness, compressive strength, and wear resistance are essential properties in clinical terms, and the quality of individual glass ionomer materials is expressed by these characteristics [19]. These mechanical properties are related to the microstructure of the material, and the wear of the material may be due to the action of mechanical, thermal, and chemical factors [20]. The determination of wear resistance is important for any restorative material used for long-term fillings, especially in the posterior segment.

The objective of the present study was to determine the wear rate of the new glass hybrid material Equia Forte HT Fil with resin coating Equia Forte Coat or without coating, and compare it with Fuji IX GP high-viscosity GIC, in conditions with acid load or at approximately neutral pH values.

## 2. Materials and Methods 

Recordings and data collecting were carried out at the Faculty of Mechanical Engineering and Naval Architecture, University of Zagreb. The research was approved by the Ethics Committee of the School of Dental Medicine in Zagreb, decision number: 05-PA-30-XVIII-6/2020.

### 2.1. Samples

The experiment was performed on two materials: the Equia Forte HT Fil glass hybrid restorative material (GC, Tokyo, Japan) and the Fuji IX GP conventional high-viscosity glass ionomer (GC Europe, Leuven, Belgium). The samples were prepared using a rectangular silicone mold with dimensions of 7 mm in diameter (d) × 20 mm in height (h). Both materials belong to encapsulated systems; thus, each capsule was placed in a 3MTM ESPETM CapMixTM mixer (3M ESPE, Seefeld, Germany) for 10 seconds during preparation, according to the manufacturer’s instructions. Immediately after mixing, the contents of the two capsules were applied to the mold and gently pushed with a celluloid strip and a smooth condenser to prevent the formation of air bubbles and to achieve a flat and smooth surface. After 5 min, the surface of each individual sample was polished with finishing paper (Standard metallographic grinding paper, P1000, (Pace Technologies, Tucson, AZ, USA), and cylindrical samples were obtained. Twenty-four randomly selected samples of Equia Forte HT Fil were coated with a protective coating and illuminated with a D-Light Duo polymerization lamp (GC, Tokyo, Japan) for 20 s. Three groups of samples (*n* = 24) were used: (i) Equia Forte HT Fil with Coat, (ii) Equia Forte HT Fil without Coat, and (iii) Fuji IX GP. The samples were stored in distilled water for 10 days at a temperature of 24 °C for the materials to completely set.

### 2.2. Exposure of Samples to Solutions of Different pH Values

After glass ionomer maturation, the samples were randomly divided into three subgroups (*n* = 8) and stored in different media (i.e., exposed to an acidic environment, as shown in Table 1). The artificial saliva used as a medium for storing samples was SAGF medium [21]. The pH value of artificial saliva was 6.75, and the acidic solution used to expose the second and the third subgroup was Coca-cola drink (Coca-Cola Company, Atlanta, GA, USA) with a pH of 2.5. 

### 2.3. Mass Recordings and Determining Wear Resistance

All the samples were weighed on an NBL 254i analytical balance (Mettler-Toledo, Greifensee, Switzerland), with a readability of four decimal places. After that, the samples were brushed in an authentic device made at the Faculty of Mechanical Engineering and Naval Architecture in Zagreb [22]. The device included an Aquafresh Clean Control toothbrush (GlaxoSmithKline, Bentford, U.K.), medium hardness. The toothbrush is attached to the upper jaw of the device, while a sample is attached to the lower jaw. After loading the sample with a weight over the lever, the toothbrush slides over the surface of the sample. The counter provides insight into the number of cycles. The brushing frequency was set at 1.25 Hz with 1N load for 5000 cycles, resulting in a total of 1.5 h of brushing. Two samples could be tested at the same time. The brush was moved in the horizontal direction with an amplitude of 5 mm. During brushing, the samples were exposed to a constant flow of a mixture of toothpaste (Aquafresh triple action, GlaxoSmithKline, Brentford, U.K.) and water, at a ratio of 1:1. After a cycle of 5000, the samples were dried with absorbent paper, left at room temperature for 15 mins, and weighed on the analytical balance. 

### 2.4. Statistical Analysis

Statistical analysis was performed by the method of one-way and two-way analysis of variance, comparing differences between sample masses before and after the procedure (SPSS v. 20, program, IBM).

## 3. Results

**Figure 1:**
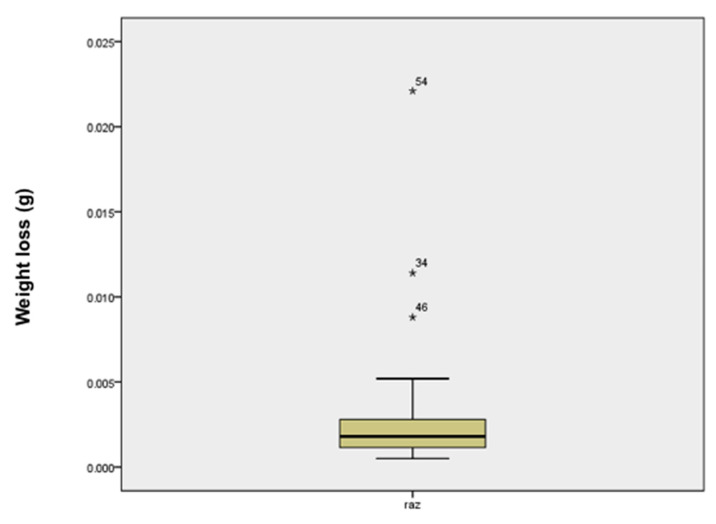
Distribution of mass differences in grams before and after brushing for all the samples. Three samples stood out and were excluded form statistical analysis.

The distribution of wear values (the differences in mass before and after brushing) is shown in Figure 1. Three samples stood out and were excluded from the analysis.

In the power analysis, the obtained effect of the Cohen effect for the comparison of materials was f = 0.879, and for the comparison of storage methods, f = 0.398. At the level of significance *p* < 0.05 and effect size f = 0.3, the calculated power for materials was *N* = 0.97, and for storage conditions, *N* = 0.998.

Statistical analysis showed that the wear resistance significantly differed depending on the material (*p* = 0.000) and acid exposure (*p* = 0.000) (Table 2).

**Figure 2:**
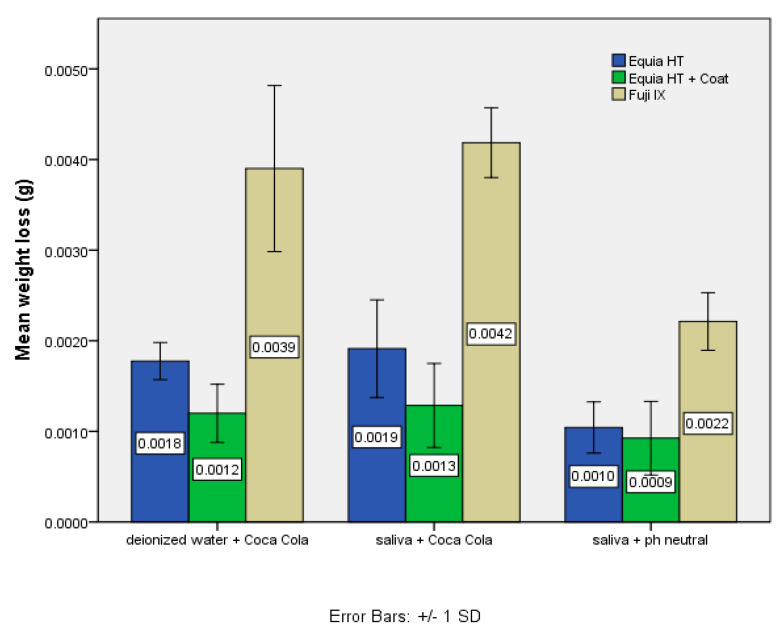
The wear (mean weight loss) of Equia Forte HT Fil with Coat was the lowest, followed by the wear of Equia Forte Fil without Coat. The highest was the wear of Fuji IX GP, considering all storage/acid exposure conditions.

Equia Forte HT Fil coated with Equia Forte Coat had the lowest weight loss, followed by Equia Forte HT Fil without coating, and the largest weight loss was recorded for the Fuji IX GP material (Figure 2). These differences apply to all three storage/acid exposure conditions. All the differences were statistically significant (*p* < 0.01).

Considering acid load—in the subgroup, where the samples were stored in distilled water and treated with acid solution —the difference was significant between Fuji IX GP and Equia Forte HT, regardless of Coat (*p* = 0.000), while the difference between Equia Forte HT and Equia Forte HT with Coat was not statistically significant (*p* = 0.158) (Table 3). In the subgroup where the samples were stored in artificial saliva and treated with acid solution, the difference was also significant between the Fuji IX GP group and the Equia Forte HT group (*p* = 0.000), and the difference between the Equia Forte HT group with Coat and Equia Forte HT without Coat was not significant (*p* = 0.058). Similarly, in the subgroup where the samples were stored in artificial saliva and not treated with acid solution, a statistically significant difference was observed between Fuji IX and the two Equia Forte HT groups (*p* = 0.000), while the Equia Forte HT groups were not statistically significantly different (*p* = 0.803) (Table 4).

The medium in which the samples were stored did not significantly affect material wear, and the differences between the samples stored in saliva and treated with acid solution and the samples stored in water and treated with acid solution were not significant (*p* = 0.588). Exposure to additional acid load significantly affected wear, and the difference between the group not exposed to acid solution and the two groups where samples were exposed to acidic environment was significant (*p* = 0.000) (Table 5).

## 4. Discussion

The results of our study showed that the wear of the glass hybrid material is significantly lower than the wear of the high-viscosity glass ionomer, under all pH conditions. The analysis within the subgroups with different acid loads showed that the coating did not significantly increase the wear resistance of the glass hybrid material, nor did the storage medium significantly affect wear. However, periodic exposure of the material to a low pH (five times for 5 min per day) has led to significantly higher wear of all the materials: high-viscosity GIC and hybrid glass with or without coating. 

In addition to compressive strength and microhardness, wear resistance is a mechanical property that is especially important in assessing the capacity of a material to resist deformation and gradual removal due to mechanical or chemical causes. It is the ability of a material to withstand the application of force while maintaining the original shape and function of the filling. In many studies, wear simulation has been achieved by brushing with a toothbrush [23,24], and with wear amount measured by weight loss [25,26]. According to Kanter and Koski [27], maintaining an oral hygiene with one daily brushing session results in 4320 brushing cycles after one year. In this study, 5000 cycles were decided. Therefore, we can conclude that our results simulate material wear within 14 months of average brushing. In this study, a vertical force of 1 N was applied, which is consistent with other studies where the average applied force for a similarly set up experiment was 1—3N (i.e., 100–400 gf) [28,29]. In the mouth, abrasion in the contact of two bodies and abrasion in the contact of three bodies mostly take place [30]. The three-body system consists of an abrasive body, antibodies, and intermediate bodies (particles) that move freely and act abrasively. Compared to a two-body system, it results in a higher value of abrasive wear. A system of three bodies was achieved by exposing the samples to a mixture of toothpaste (“Aquafresh” triple action) and water at a ratio of 1:1.

In the context of the 2013 Minamata Convention on the Reduced Use of Mercury [31], a cost-effective and clinically suitable mercury free alternative to dental amalgam is needed for its replacement. Glass ionomers are already proven as clinically acceptable materials, but their mechanical properties need some improvement to satisfy requirements for long-lasting filling material. Therefore, the research and development of glass ionomers over the decades has been focused on improving the mechanical properties of GIC materials. Significantly better clinical performance than its predecessors was achieved by the high-viscosity GIC “Equia Fil” material, which was coated with the nano-filled resin coating “Equia Coat”, and the microlaminated technique achieved significantly better mechanical properties compared to conventional cements. The manufacturer presented the Equia material as a durable material for first-class fillings and small two-plane fillings of the second class in the lateral region [13,14]. Combined with the microlaminated technique, the development of GIC materials was followed by modifications to the filler by adding small, more reactive glass particles, resulting in the introduction of the Equia Forte material in 2015 and the latest Equia Forte HT material in 2019. The last modification, was, according to the manufacturer, the inclusion of particles and fillers with close refractive indexes, resulting in increased translucency and enhanced aesthetic properties of the material.

The immediate predecessor of the Equia Forte HT used in our study, Equia Forte Fil with the Equia Forte Coat, is a relatively new material, but it has been on the market long enough that numerous studies have been conducted that largely confirm the published mechanical properties. Thus, Cosgun et al. [32] proved in their study that Equia Forte has a higher nanohardness compared to Fuji IX. The samples were stored in distilled water at 37 degrees, and the hardness was tested by a mechanical device that applied a constant force per unit area. In a similar study, Moshaverinia et al. [33] concluded that the microhardness of Equia Forte is higher than the microhardness of Fuji IX. Hardness and wear resistance are proportional [34]. Therefore, we can conclude that these studies support our results. Following the above, Abdullah Saleh Al Jamhan [35] tested and compared the wear resistance of several glass ionomers. The results showed that the Equia Forte Fil has higher wear resistance than the new generation Fuji IX (Fuji IX GP Extra) and the Fuji IX. Our results are in line with the above results: the Equia Forte HT glass hybrid material has proven to be more wear resistant than the Fuji IX high-viscosity glass ionomer material.

We have already mentioned the laminated technique (i.e., increasing the abrasion resistance through the application of a resin-based nano-filled coating) used with a variety of products, from the Fuji IX GP Extra from 2007 to the newer glass hybrid materials with associated coatings. This coating (i.e., Coat) does not have the simple function of protecting against excess water in the maturation phase of the glass ionomer, but it actively contributes to the hardness of the material and abrasion resistance [36,37]. This effect is attributed to the penetration of the resin through the surface of the GIC and the filling of cracks and porosity [37]. Indeed, previous researchers have shown that the Equia Forte Fil without coating Equia Forte Coat has poorer physical properties [38,39,40]. These conclusions are consistent with our results; however, this difference was not statistically significant in our study. In their in vitro study, Kanik et al. [38] concluded that a coating made of Equia Forte Fil with a coating has higher wear resistance after a cycle of 7500 brushes compared to one without a coating; however, the situation was reversed after 2500 brushes. In a similar study, Wang et al. [41] concluded that the filling strength of Equia Forte Fil with or without coating is almost indistinguishable. This is explained by the fact that Coat protects material from wear and tear by consuming itself [38]. However, after a cycle of 20,000 brushes, the Coat is completely worn out, and it no longer exists on the filling surface [38]. Nevertheless, its protective effect in the maturation phase of the material can contribute to an increase in long-lasting mechanical properties even after the Coat is consumed. Namely, in maturation, the Coat protects the material from excessive water and the loss of ions that actively participate in the formation of ionic bonds with the carboxyl groups of polyacrylic acid, which can indirectly affect the mechanical properties of the material [42]. Our results suggest that the Coat has a positive effect on wear resistance, but not significantly, and the explanation for the significant wear resistance of Equia Forte HT compared to Fuji IX in all pH conditions must be sought in the composition of the material. Highly reactive small particles probably add to the standard glass ionomer filler in Equia Forte HT materials and improve the mechanical properties by facilitating and accelerating the formation of chemical bonds between the filler particles and the organic acid (i.e., the liquid component of the material), which then leads to improved mechanical properties. The pH of the solution to which the material is exposed has an additional effect on wear resistance. The mass loss values after a cycle of 5000 brushes were highest for the samples stored in artificial saliva and treated with acid, while for the samples stored in distilled water and treated with acid, the values were slightly lower, but not significantly. This result is not consistent with the results of a study by Poornima et al. [43], which showed that the compressive strength and microhardness of Equia Forte with coating and conventional GIC are significantly affected by the medium in which the samples are stored. There were several other reports where the highest values were recorded in the samples stored in distilled water, followed by artificial saliva, while the compressive strength and microhardness were the lowest in samples stored in acid after 7 and 30 days [44,45]. Although the results of the present study follow this trend, the wear resistance was not significantly higher in distilled water compared to artificial saliva. This can be explained by short-term exposure of the storage media to air in this study. On the one hand, distilled water absorbs CO_2_ from the air and reacts with it to form carboxylic acid, resulting in slightly acidic pH. On the other hand, the loss of hydrogenocarbonates from artificial saliva, because of the high vapor pressure of CO_2_, raises the pH of the artificial saliva during short-term exposure [46]. Furthermore, the present results also reveal how periodic exposure to a low pH solution alone influences some GIC materials, regardless of the medium in which the samples were stored, and again, Equia Forte HT performed better. This is in agreement with Perera et al. [47], finding that the more recent GIC materials (GC Fuji Bulk and GC Equia Forte Fil) showed increased acid resistance over the older GIC materials, including Fuji IX. The enhanced formulation of the new generation highly viscous GICs and glass hybrid materials increases strength and acid resistance [47]. 

Advances in technology and interdisciplinarity have led to the application of stem cells and tissue engineering in dentistry, enabling the regeneration of bone, periodontium, and other oral tissues, including mineralized dental tissues [10,11]. Nevertheless, the regeneration of enamel and dentine by targeted stem cell differentiation is not likely to be a clinical practice soon, and the improvement of bio-mechanical properties of restorative materials is of significant clinical importance. Considering acidic load and wear resistance, the performance of glass hybrid material Equia Forte HT Fill was significantly better compared to high viscosity GIC. 

## 5. Conclusions

Within the limitations of this study, we can conclude that:

1. The glass hybrid material Equia Forte Fil HT is more resistant to wear than the high-viscosity GIC Fuji IX GP in all storage conditions and acid load.
2. Equia Forte Fil HT samples coated with Equia Forte Coat are more resistant to brush-induced wear compared to samples not coated with Coat, but not significantly.
3. The storage medium (distilled water or artificial saliva) does not significantly affect the wear of the Fuji IX GP and Equia Forte HT Fil materials.
4. The wear of material is significantly higher with periodic exposure to a low pH solution, independent of the storage medium.

## Figures and Tables

**Table 1:** Equia Forte HT Fil groups with and without Coat and Fuji IX Group were divided into three subgroups depending on the storage medium and exposure to a low pH solution.

Experimental Subgroups	Storage Conditions and Exposure to a Low pH Solution
Subgroup 1	The samples were stored for 7 days in artificial saliva.
Subgroup 2	The samples were stored for 7 days in artificial saliva and exposed to acidic environment five times for 5 minutes per day.
Subgroup 3	The samples were stored for 7 days in distilled water and exposed to acidic environment five times for 5 minutes per day.

**Table 2:** Analysis of variance showed a difference in wear in relation to the material, acid load, and the combination of material and acid load (acid * material).

Source	Type III Sum of Squares	Df	Mean Square	F	Sig.
Corrected Model	8.737 × 10^−5 a^	8	1.092 × 10^−5^	48.748	<0.00005
Intercept	<0.00005	1	<0.00005	1287.735	<0.00005
Acid	1.498 × 10^−5^	2	7.492 × 10^−6^	33.440	<0.00005
Material	6.803 × 10^−5^	2	3.402 × 10^−5^	151.836	<0.00005
Acid * material	6.243 × 10^−6^	4	1.561 × 10^−6^	6.966	<0.00005
Error	1.344 × 10^−5^	60	2.240 × 10^−7^		
Total	<0.00005	69			
Corrected Total	<0.00005	68			

a. R Squared = 0.867 (Adjusted R Squared = 0.849).

**Table 3:** Analysis of the effect of acid load on the wear of samples stored in distilled water.

(I) Materialb	(J) Materialb	Mean Difference (I-J)	Std. Error	Sig.	95% Confidence Interval	
					Lower Bound	Upper Bound
Equia HT	Equia HT + Coat	0.5750	0.0002865	0.158	−0.000179	0.001329
	Fuji IX	−0.0021250 *	0.0002865	0.000	−0.002879	−0.001371
Equia HT + Coat	Equia HT	−0.0005750	0.0002865	0.158	−0.001329	0.000179
	Fuji IX	−0.0027000 *	0.0002865	0.000	−0.003454	−0.001946
Fuji IX	Equia HT	0.0021250 *	0.0002865	0.000	0.001371	0.002879
	Equia HT + Coat	0.0027000 *	0.0002865	0.000	0.001946	0.003454

* The mean difference is significant at the 0.05 level.

**Table 4:** Analysis of the effect of acid load on the wear of samples stored in artificial saliva.

(I) Materialb	(J) Materialb	Mean Difference (I-J)	Std. Error	Sig.	95% Confidence Interval	
					Lower Bound	Upper Bound
Equia HT	Equia HT + Coat	0.0001179	0.0001771	0.803	−0.000350	0.000586
	Fuji IX	−0.0011696 *	0.0001771	0.000	−0.001638	−0.000702
Equia HT + Coat	Equia HT	−0.0001179	0.0001771	0.803	−0.000586	0.000350
	Fuji IX	−0.0012875 *	0.0001711	0.000	−0.001740	−0.000835
Fuji IX	Equia HT	0.0011696 *	0.0001771	0.000	0.000702	0.001638
	Equia HT + Coat	0.0012875 *	0.0001711	0.000	0.000835	0.001740

* The mean difference is significant at the 0.05 level.

**Table 5:** Analysis of the influence of storage media and additional acid load on the wear of the samples.

	(I) Acid Load	(J) Acid Load	Mean Difference (I-J)	Std. Error	Sig.	95% Confidence Interval	
						Lower Bound	Upper Bound
Scheffe	deionized water + acid solution	Saliva + acid solution	−0.000145	0.0001397	0.588	−0.000495	0.000206
		saliva-pH neutral	0.000883 *	0.0001381	0.000	0.000536	0.001230
	saliva + acid solution	deionized water + acid solution	0.000145	0.0001397	0.588	−0.000206	0.000495
		saliva-pH neutral	0.001028 *	0.0001412	0.000	0.000673	0.001382
	saliva-ph neutral	deionized water + acid solution	−0.000883 *	0.0001381	0.000	−0.001230	−0.000536
		saliva+acid solution	−0.001028 *	0.0001412	0.000	−0.001382	−0.000673

Based on observed means. The error term is Mean Square (Error) = 2.240 × 10^−7^. * The mean difference is significant at the 0.05 level.

## Data Availability

The data that support the findings of this study are available from the corresponding author upon request.
